# Tumoral immune-infiltrate (IF), PD-L1 expression and role of CD8/TIA-1 lymphocytes in localized osteosarcoma patients treated within protocol ISG-OS1

**DOI:** 10.18632/oncotarget.22912

**Published:** 2017-12-04

**Authors:** Emanuela Palmerini, Claudio Agostinelli, Piero Picci, Stefano Pileri, Teresa Marafioti, Pier-Luigi Lollini, Katia Scotlandi, Alessandra Longhi, Maria Serena Benassi, Stefano Ferrari

**Affiliations:** ^1^ Chemotherapy Unit, IRCCS, Istituto Ortopedico Rizzoli, Bologna, Italy; ^2^ Haematopathology Unit, Policlinico S. Orsola-Malpighi, Bologna, Italy; ^3^ Haematopathology Unit, European Institute of Oncology, Milan, Italy; ^4^ Bologna University School of Medicine, Bologna, Italy; ^5^ University College London Cancer Institute, London, United Kingdom; ^6^ Department of Experimental, Diagnostic and Specialty Medicine (DIMES), University of Bologna, Bologna, Italy

**Keywords:** osteosarcoma, PD-1, PD-L1, CD8, tumor microenvironment

## Abstract

**Background:**

We hypothesized that immune-infiltrates were associated with superior survival, and examined a primary osteosarcoma tissue microarrays (TMAs) to test this hypothesis.

**Methods:**

129 patients (pts) with localized osteosarcoma treated within protocol ISG-OS1 were included in the study. Clinical characteristics, expression of CD8, CD3, FOXP3, CD20, CD68/CD163 (tumor associated macrophage, TAM), Tia-1 (cytotoxic T cell), CD303 (plasmacytoid dendritic cells: pDC), Arginase-1 (myeloid derived suppressor cells: MDSC), PD-1 on immune-cells (IC), and PD-L1 on tumoral cells (TC) and IC were analysed and correlated with outcome.

**Results:**

Most of the cases presented tumor infiltrating lymphocytes (TILs) (CD3+ 90%; CD8+ 86%). Tia-1 was detected in 73% of the samples. PD-L1 expression was found in 14% patients in IC and 0% in TC; 22% showed PD-1 expression in IC.

With a median follow-up of 8 years (range 1-13), the 5-year overall survival (5-year OS) was 74% (95% CI 64-85). Univariate analysis showed better 5-year OS for: a) pts with a good histologic response to neoadjuvant chemotherapy (p = 0.0001); b) pts with CD8/Tia1 tumoral infiltrates (p = 0.002); c) pts with normal alkaline phosphatas (sALP) (p = 0.04). After multivariate analysis, histologic response (p = 0.007) and CD8/Tia1 infiltration (p = 0.01) were independently correlated with survival. In the subset of pts with CD8+ infiltrate, worse (p 0.02) OS was observed for PD-L1(IC)+ cases.

**Conclusions:**

Our findings support the hypothesis that CD8/Tia1 infiltrate in tumor microenvironment at diagnosis confers superior survival for pts with localized osteosarcoma, while PD-L1 expression is associated with worse survival.

## INTRODUCTION

Ostesarcoma is a rare, aggressive sarcoma. Whereas there is an agreement that surgery, with adjuvant chemotherapy, is paramount for the primary therapy of the cancer, about 30% of patients without evident metastases at presentation will die of disease [1]. The prognosis is even poorer (10% survival at 5-years) in patients with synchronous metastases [1]. Therefore there is an urgent need to identify new targets, different risk groups and predictive factors for tailored treatment for each individual.

Various mechanisms have been proposed for the resistance of human solid tumors to immune recognition and obliteration, including the recruitment of regulatory T cells (T-reg), myeloid derived suppressor cells (MDSC) [2] and up-regulation of immune inhibitory ligands, such as the ligand of programmed cell death protein 1 (PD-L1) [3].

T-lymphocyte and antigen-presenting cells (APCs) interaction is in fact bi-directional and mediated by PD-L1 and programmed cell death protein 1 (PD1) on APCs and on lymphocytes. PD-L1 is expressed also by several tumors [3].

**Figure 1:**
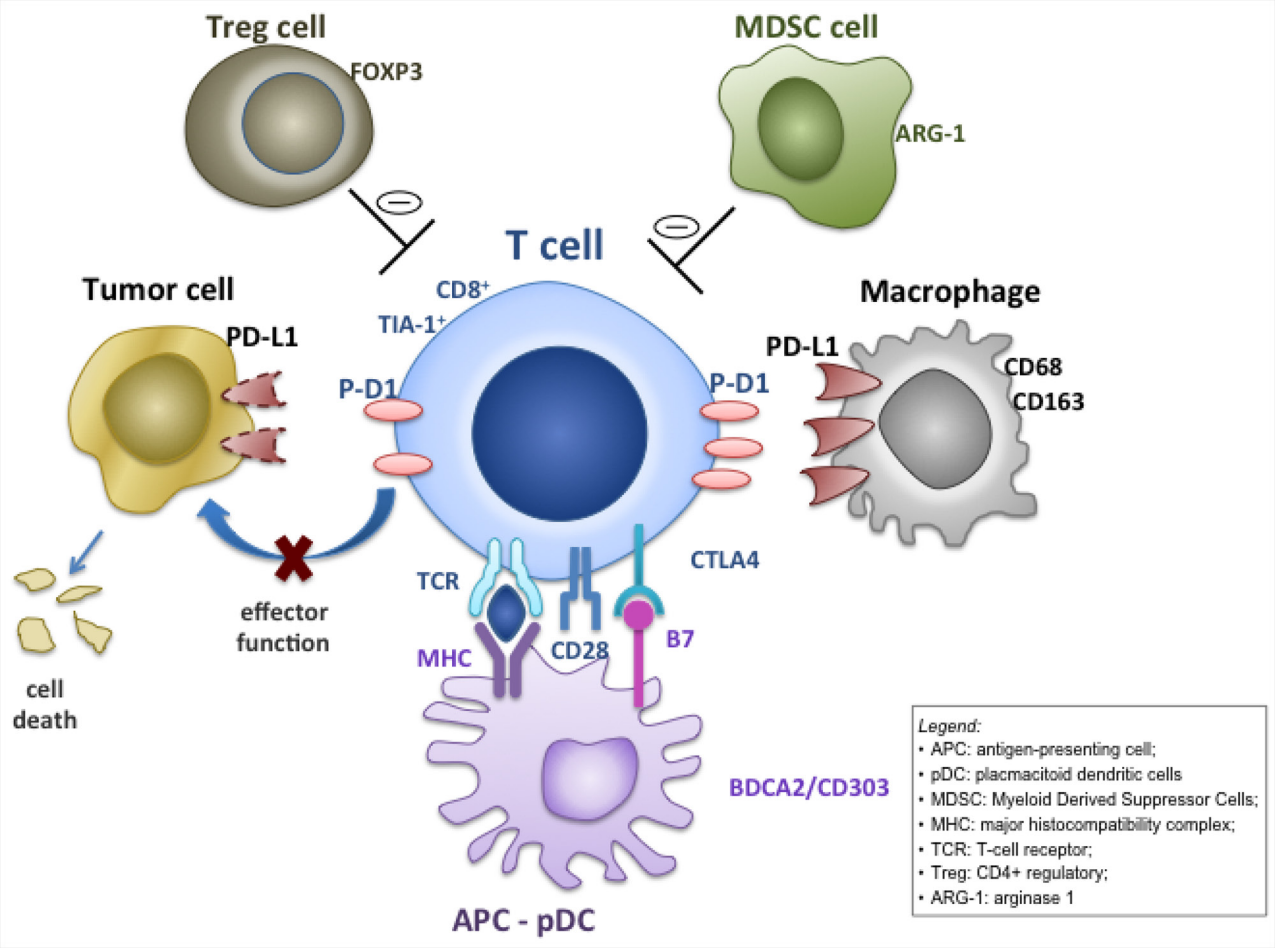
Mechanisms for intratumoral programmed cell death ligand-1 (PD-L1) expression Adaptive focal expression of PD-L1 by macrophages (CD68^+^/CD163^+^) occurs at the interface of tumor cell nests with immune infiltrates secreting pro-inflammatory factors such as interferon-γ. The ligation of PD-L1 on macrophage and, in some histotypes, on tumor cells, with programmed cell death protein 1 (PD-1) molecules will down-modulate T cell function, essentially creating a negative feedback loop that reduces antitumor immunity (the so called ‘tumor shield’ effect), eventually reducing CD8 tumoricidal function.

PD1/PD-L1 pathway inhibitors such as pembrolizumab, nivolumab, atezolizumab have been approved from several solid tumors (melanoma, kidney, lung cancer, head and neck, Merkel cell tumors, Hodgkin’s lymphoma), and PD-L1 expression on tumoral cells (TC) or in immune-infiltrate (IC) has been associated with response to checkpoint inhibitors, in some cases [4], but PD-L1 expression predictive value remain controversial [5, 6]. Recently, anecdotal responses with pembrolizumab, nivolumab and also with nivolumab/pazopanib combination were reported in osteosarcoma patients [7–9]. These compounds interrupt PD-1/PD-L1 axis, impeding the PD-L1 mediated ‘tumor shield’ effect (Figure 1).

Few data on osteosarcoma microenvironment composition and the PD-1/PD-L1 expression are available, and most of the studies refer to small series with incomplete clinical information, inhomogeneous for stage (metastatic and localized patients), treatments and timing of biopsies [10].

The present study aim is to characterize the immune-infiltrates and PD-L1/PD-1 pathway in sample of chemo-naïve patients with localized osteosarcoma, treated according to the same protocol, in order to assess its prognostic implications and its potential role in cancer immune-evasion.

## RESULTS

### Tumoral microenvironment components prior chemotherapy (bioptic samples)

Eighty-six out of 129 cases analysed were evaluable for at least 7 markers and were included in the present study.

**Figure 2:**
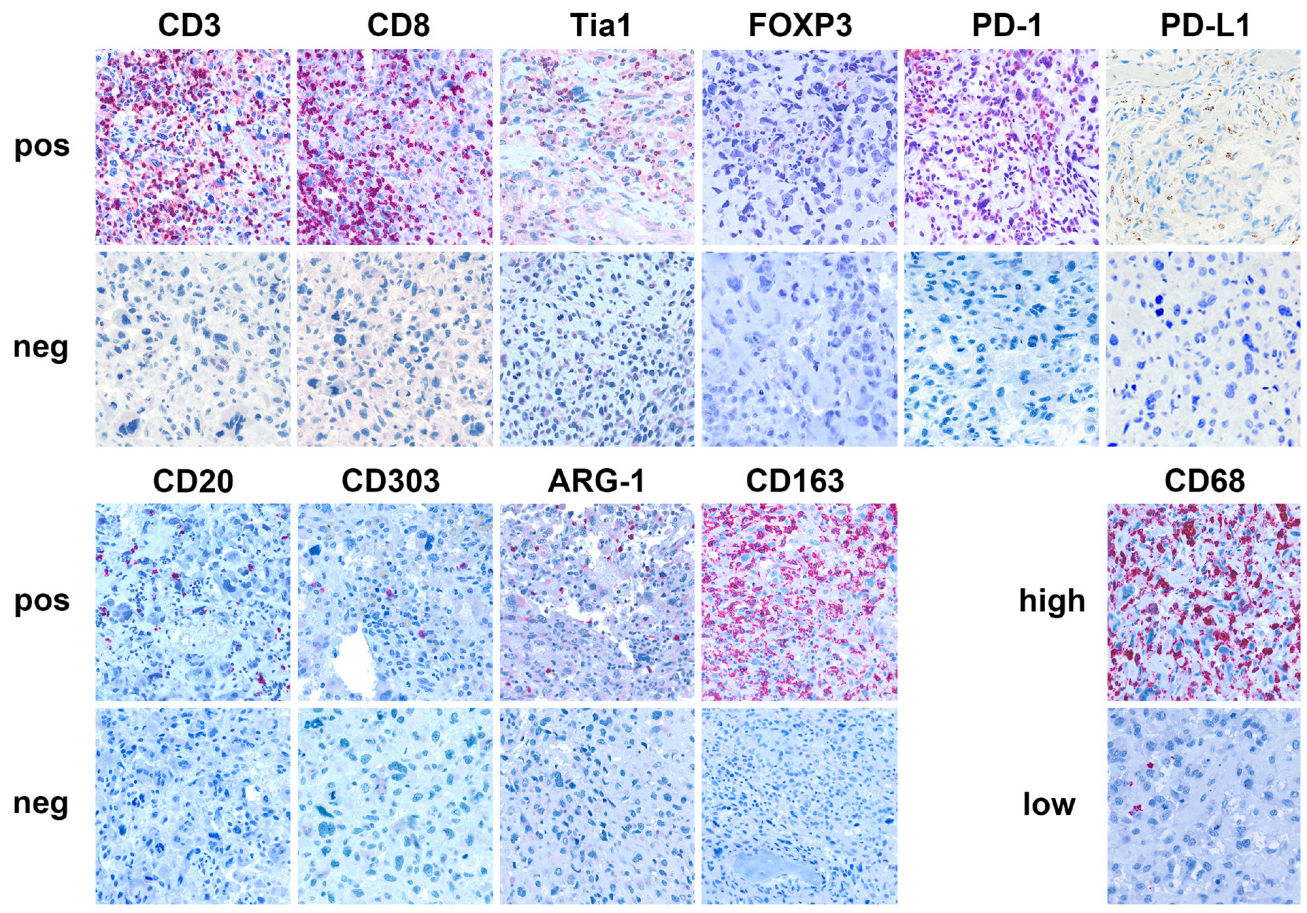
Tumor microenvironment in localized osteosarcoma Immunohistochemical expression of CD3, CD8, CD20 (T tumor infiltrating lymphocytes: TILs), Tia-1 (cytotoxic T cell), FOXP3 (T regulatory lymphocytes: T-regs), PD-1, PD-L1, CD68 (tumor associated macrophages: TAM), BDCA-2/CD303 (plasmacytoid dendritic cells: pDC), Arginase-1 (myeloid derived suppressor cells: MDSC) proteins.

Most of the cases presented TILs (CD3+ 77/86, 90%; CD8+ 74/86, 86%, CD20+ 25/86, 29%), while FOXP3^+^ (Treg) were detected in 28/86 (33%) of the patients (Table 1; Figure 2). Tia-1 was detected in 57/78 (73%) of the samples. TAMs (CD163 positive) were observed in the microenvironment in 47/70 (67%) of the patients, while 31/74 (37%) patients presented also high levels of CD68 positivity (Table 1; Figure 2). Only 3/78 (4%) and 16/78 (21%) cases presented CD303+ and Arginase-1+ cells. PD-L1 expression was found in 12/86 (14%) patients in IC and 0/86 (0%) in TC; 19/86 (22%) showed PD-1 expression in IC (Table 1; Figure 2).

**Table 1:** Immunological characterization of tumor microenvironment in 86 patients with localized osteosarcoma

	CD3 n (%)	CD8 n (%)	CD20 n (%)	FOXP3 n (%)	Tia1 n (%)	CD68^*^ n (%)	CD163 n (%)	CD303 n (%)	Arg-1 n (%)	PD-L1 (TC) n (%)	PD-1 (IC) n (%)	PD-L1 (IC) n (%)
**Pos**	77 (90)	74 (86)	25 (29)	28 (33)	57 (73)	31 (37)	47 (67)	3 (4)	16 (21)	0 (0)	19 (22)	12 (14)
**Neg**	9 (10)	12 (14)	61 (71)	58 (67)	21 (27)	53 (63)	23 (33)	75 (96)	62 (79)	86 (100)	67 (78)	74 (86)

Arg-1: Arginase-1; TC: tumor cells; IC: immune cells; ^*^ CD-68: Pos: high level expression; neg: low level expression.

### Survival analysis

**Figure 3:**
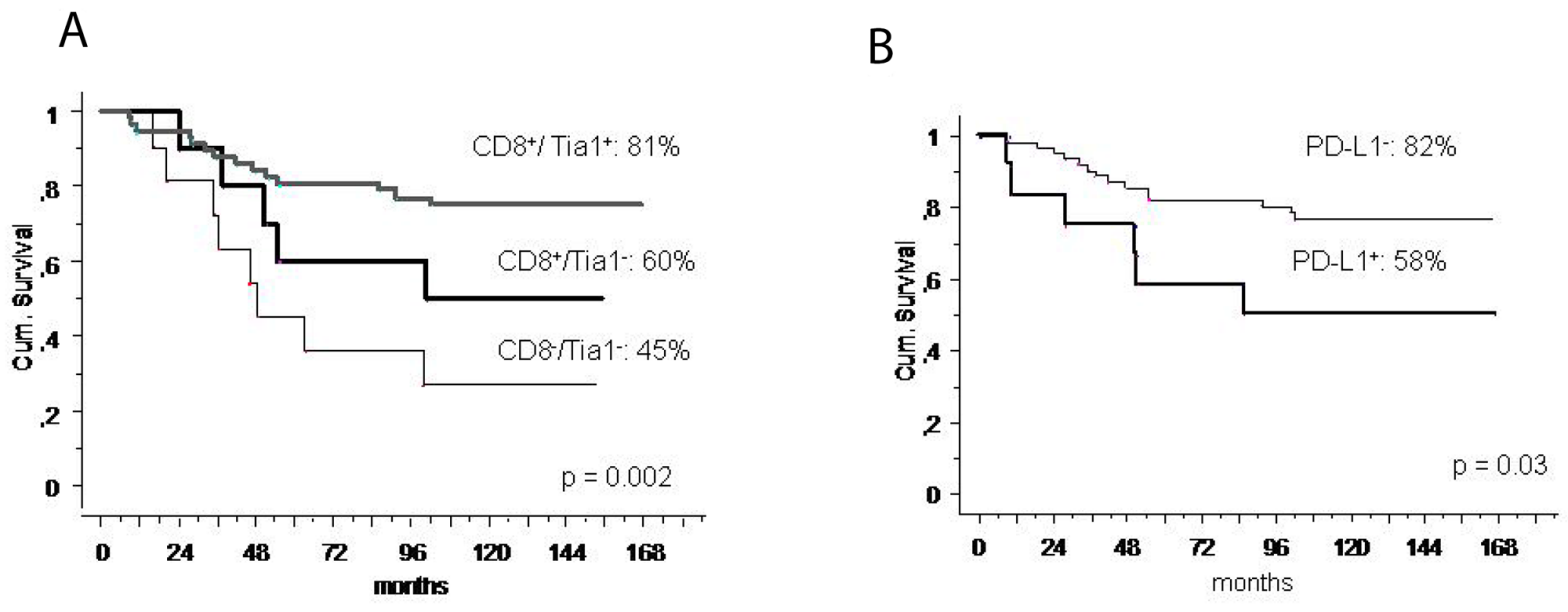
Survival and immune-infiltrate **(A)** 5-year overall survival according to CD8/Tia1 expression in localized osteosarcoma; (**B)** 5-year overall survival according to PD-L1 expression in patients with CD8+ localized osteosarcoma.

With a median follow-up of 8 years (range 1-13), the 5-year OS was 74% (95% CI 64-85). Univariate analysis showed better 5-year OS for good responders (good 89% vs poor 57%, *p* = 0.0001), for cases with CD8/Tia1 tumoral infiltrates (CD8^+^/Tia1^+^ 81% vs CD8^+^/Tia1^-^ 60% vs CD8^-^/Tia1^-^ 45%, *p* = 0.002) and patients with normal AP at baseline (AP normal 85% vs AP high 44%, *p* = 0.04) (Table 2, Figure 3). No statistically significant difference was observed in 5-year OS according to PD-1, FOXP3, CD68, CD20, Arginase-1, CD303, CD163 expression in microenvironment, and age, gender or LDH, while PD-L1 (IC) positive cases had a non-significant inferior 5-year OS (PD-L1^+^ 58% vs PD-L1^-^ 77%, *p* = 0.14) (Table 2).

**Table 2:** Univariate Analysis for Overall Survival (OS) in patients with localized osteosarcoma

	Pts N.	% 5-year OS	95% CI	P-value
**Overall**	86	74.5	65-84	
**Age**				**0.9**
≥ 18 years	27	74	57-90	
< 18 years	59	75	63-86	
**Sex**				**0.8**
Female	33	76	61-90	
Male	53	74	61-89	
**Histologic Response°**				**0.0001**
Good	45	89	80-98	
Poor	40	57	42-73	
**CD8**				**0.003**
Positive	74	78	69-88	
Negative	12	50	22-78	
**TIA-1 °°**				**0.008**
Moderate	14	86	67-100	
Focal	43	79	67-91	
None	21	52	31-74	
**CD8/ Tia1 °°**				**0.002**
Positive / Positive	57	81	70-91	
Positive / Negative	10	60	30-90	
Negative / Negative	11	45	16-75	
**CD3**				**0.07**
Positive	77	78	69-87	
Negative	9	44	12-77	
**FOXP3** ^*^				**0.13**
Positive	28	75	54-93	
Negative	56	73	64-85	
**PD-1 (IC)**				**0.6**
Positive	19	74	54-93	
Negative	67	74	64-85	
**PD-L1 (IC)**				**0.14**
Positive	12	58	30-86	
Negative	74	77	67-87	
**LDH °°**				**0.15**
Normal	60	78	68-89	
High	18	61	39-84	
**sAP**				**0.04**
Normal	48	85	75-95	
High	38	64	48-80	
**Arginase-1** ^**^				**0.3**
Positive	16	81	62-100	
Negative	61	70	59-82	
**CD303 °°**				**0.9**
Positive	3	67	13-100	
Negative	75	73	63-83	
**CD68** ^*^				**0.1**
High	31	84	71-99	
Low	53	67	54-80	
**CD163** ^***^				**0.17**
Positive	47	81	70-92	
Negative	23	56	36-77	

° Not available in 1 patient; °° non available in 8 patients; ^*^ not available in 2 patients; ^**^ not available in 7 patients; ^***^ not available in 16 patients

After multivariate analysis, good histologic response (p = 0.007) and a CD8^+/^Tia1^+^ lymphocytic infiltrate (0.05) were independently associated with better survival (Table 3).

**Table 3:** Multivariate Analysis for Overall Survival (OS) in patients with localized osteosarcoma

Variable	RR	95% CI	P
**CD8/Tia1**			
Positive/Positive	1		**0.05**
Positive/Negative	1.8	0.7-5.2	**0.2**
Negative/Negative	3.1	1.2-7.8	**0.01**
**Histologic Response**			
Poor	1		**0.007**
Good	0.27	0.1-0.7	
**sPA**			
Normal	1		**0.2**
High	1.75	0.8-3.9	

Since PD-L1(IC) is a marker of cytotoxic function exhaustion, we investigated if PD-L1 expression could influence survival in the subgroup of patient with CD8^+^ lymphocytes: at univariate analysis the 5-year OS was 82% (95%CI 73%-92%) in case of PD-L1 negative and 58% (95% CI 34%-78%) in positive PD-L1 (*p* = 0.03) (Figure 3). At the multivariate analysis in this subgroup, lack of PD-L1 was and independent prognostic factor for longer survival (*p* = 0.04) (Table 4).

**Table 4:** Multivariate Analysis for Overall Survival in patients with CD8^+^ localized osteosarcoma

Variable	RR	95% CI	P
**PD-L1**			
Negative	1		**0.04**
Positive	2.8	1-7.4	
**Histologic Response**			
Poor	1		**0.02**
Good	0.3	0.1-0.9	
**sPA**			
Normal	1		**0.5**
High	1.4	0.5-3.8	

### Tumoral microenvironment components post-induction chemotherapy (surgical samples)

Based on multivariate analysis results on pre-treatment samples, a post-hoc analysis on CD8 and Tia1 was performed.

Due to post-treatment changes 33/86 patients were assessable after treatment (excluding 53 patients with massive necrosis and no tumor).

Chemotherapy-induced changes of CD8^+^ TILs were as follow: all patients with score 0 (5 cases) were unchanged, the proportion of patients with score 1 decreased: 19/33 (58%) pre-chemotherapy, 14/33 (42%) post-treatment; and the proportion of patients with a score 2/3 increased: 9/33 (27%) to 12/33 (36%) (p 0.5).

A survival analysis according to presence of CD8^+^ /Tia1^+^ infiltrates presence in the surgical samples after induction-chemotherapy, confirmed their prognostic role: 5- year OS was 78% (CI% 51-100) for CD8^+^/Tia1^+^ (22 patients), 64% (CI%43-84) for CD8^+^/Tia1^-^ (9 patients) and none was alive with CD8^-^/Tia1^-^ (2 patients) (p 0.05) (Supplementary Figure 1).

## DISCUSSION

The role of microenvironment in tumor immune escape is well recognized. Several studies addressed the role of tumor microenvironment in pathobiology and its impact on survival of osteosarcoma [11–15], however none is specific for osteosarcoma in the localized stage, nor compared the prognostic power of immune-infiltrate with the other validate prognostic factor routinely used in clinical practice [16, 17],

The present study, including patients with osteosarcoma in a localized stage, and treated within the same protocol [16], demonstrated an independent prognostic role of CD8^+^ and Tia1 lymphocytes.

The first study demonstrating the association between CD8 infiltrate and cancer specific survival was published in 2001 [18], and similar findings were subsequently confirmed among many histotypes [19, 20].

In the sarcoma field both a series of 33 ostesarcoma, including metastastic and localised patients [11], and a large soft tissue sarcoma French study, including several histotypes and also low grades lesions, failed to demonstrate a prognostic role for CD8+ lymphocytes [21].

The results of our study are in contrast with those data [11, 21], in fact a strong advantage in terms of survival has been observed in patients with CD8^+^ infiltrate.

To investigate the differentiation state of tumor-infiltrating T cells, tissues were analyzed for Tia1 expression, which is a marker of cytotoxic function: the prognostic significance of CD8^+^ cells was even more relevant when the tumor infiltrate was characterized by the concomitant presence of Tia1^+^ lymphocytes. Tia1^+^ lymphocytes might represent a more efficient subset of CD8^+^ effector cells, playing an important role in immune-surveillance of osteosarcoma [22].

The prognostic role of cytotoxic TILs CD8/Tia1 was also confirmed after induction chemotherapy, while chemotherapy does not seem to induce significant changes in CD8^+^ TILs: all cases with no CD8^+^ TILs prior chemotherapy, were confirmed negative after chemotherapy, while a slight increase on score severity (1 to 2/3) was observed.

In our study, the rate of PD-L1 expression in IC was 14%. Previous studies on osteosarcoma samples reported a higher rate of positive expression of PD-L1(IC) ranging from 25% [11] to 74% [10]. This difference might be related to the heterogeneity of the cases examined, being many of them metastatic, while our patients all had localized disease. In fact, it is well known that PD-L1 expression increases in advanced stages of the diseases as reported by Sundara Y et al: 13% in primary tumours, 25% in local relapses tissue and 48% in metastatic (*p* = 0.002) [12].

In our study a trend towards an inferior survival for positive PD-L1(IC) patients was observed. Other reports were able to demonstrate a significantly inferior event-free-survival (EFS) for osteosarcoma patients with positive PD-L1(IC) [11]. A prognostic role for PD-L1 was also confirmed by Kim et al, at RNA level [10]. Interestingly, in our series, the PD-L1(IC) expression has a prognostic significance at multivariate analysis in the subgroup of patients with CD8^+^ immune-infiltrate. It might be hypothesised that “PD-L1”-mediated immune-suppression negatively influences CD8^+^ lymphocytes function (Figure 1). In addition, it was shown that tumor response to PD-L1 or PD-1 inhibition is directly related to both the level of PD-L1 expression and lymphocytic infiltration of the tumor [23–25].

None of the patients had PD-L1 on tumor cells, as shown for other histotypes, such as colon rectal and gastric carcinoma [26, 27]. About 7% of osteosarcoma presented PD-L1 in the neoplastic clone in the study by Koirala et al [11], while head and neck squamous cell carcinoma, melanoma, breast and kidney cancer frequently express PD-L1 on the surface of tumour [4, 28]. Such variable expression among different studies may reflect the variable susceptibility of tumour cells and infiltrating immune cells to cytokines and other stromal factors in the tumour milieu [5]. In fact there are two distinct type of PD-L1 expression: the first is a constitutive (innate) expression on tumoral cells membranes, with an homogenous patter; the second is adaptative, and can be found both on tumoral cells or in macrophage [5].

In 67% of the cases we found CD163^+^ macrophages in microenvironment. CD163 was shown to be a useful marker for M2-like macrophages, which have a “pro-tumoral” activity, in contrast with M1-like macrophages characterized by a “tumoricidal” activity [29].

In our series no difference in survival according with presence of TAMs, characterized by both CD163 and CD68 expression, was observed (CD163^+^ 81% vs CD163^-^ 56%, *p* = 0.17), while in other osteosarcoma series a prognostic role of TAMs was suggested [13, 14]. This might be explained by different statistical analysis design and different stages of patients included [13, 14].

Our data suggest a role of immune-infiltrate in progression of localized osteosarcoma, and might support the use of immune-modulating agents in the treatment of this tumor. Of interest, mifamurtide, a modulator of innate immunity, which increases a wide variety of immunomodulatory molecules [15] and favours CD8 and NK cell activation [30], has been approved by EMA for the treatment of patients with localized high-grade osteosarcoma based on the results of a randomized trial [31].

**Figure 4:**
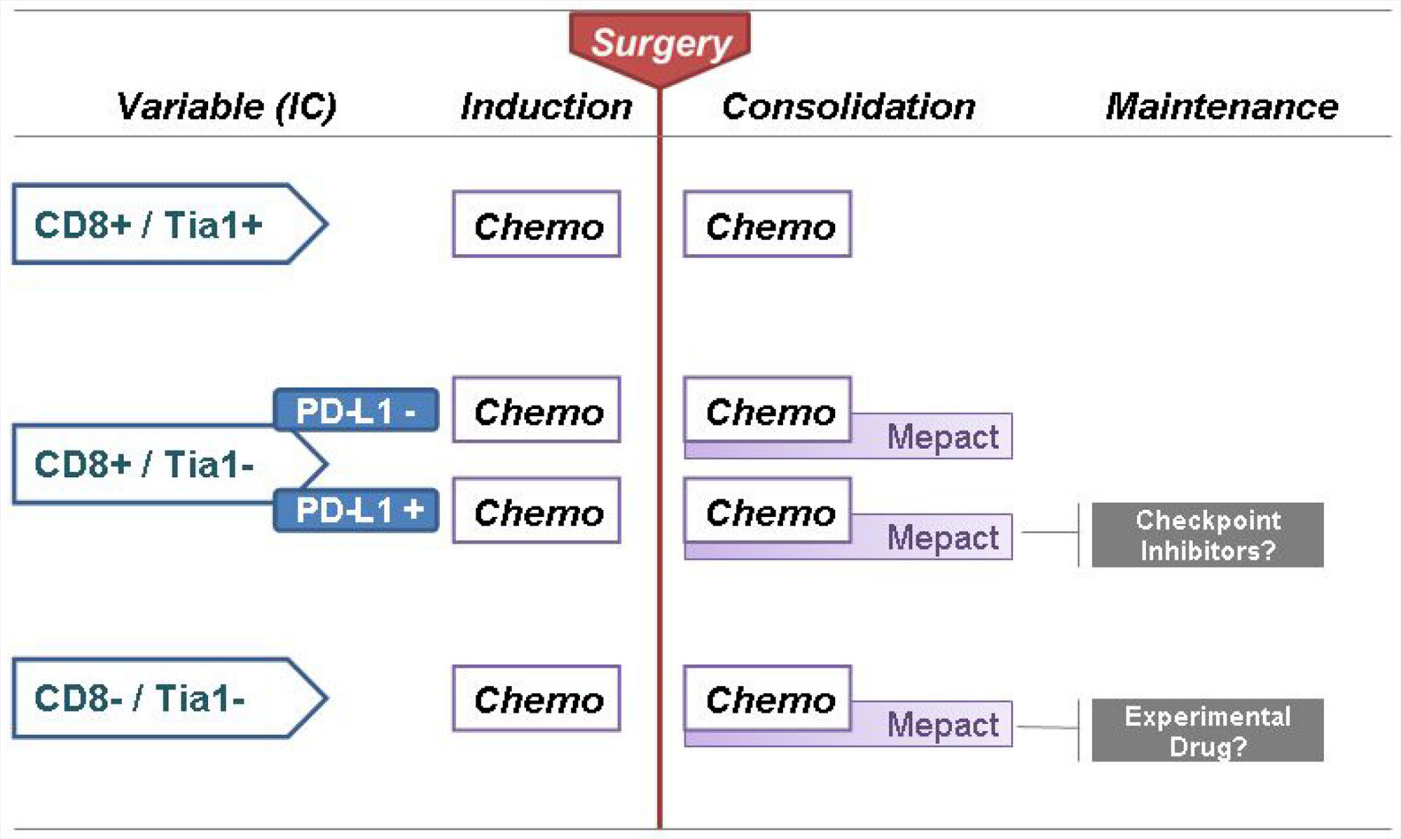
Proposal for ‘Immune-infiltrate based treatment algorithm’ for localized osteosarcoma

In conclusion, CD8/Tia1 citotoxic T-lymphocytes emerge as an important player in anti-tumor immune response. Also, this study highlights the prognostic role of tumor microenvironment in the setting of localized osteosarcoma. The data are interesting and intriguing, but a clinical application requires confirmations in other series (Figure 4).

## MATERIALS AND METHODS

### Pre-treatment (bioptic samples)

After EC approval, tissue samples obtained from biopsies performed prior chemotherapy in 129 patients were collected. Patients were prospectively treated at Istituto Ortopedico Rizzoli from 04/2001 to 11/2006 within protocol ISG-OS1, with surgery and a chemotherapy based on methotrexate, cispaltin, adriamicyn and ifosfamide as described [16].

The clinical characteristics of the patients were the following: the median age was 16 years (range 4-39 years), with paediatric patients (59/86, 67%) and male gender (53/86, 62%) being most represented. High LDH levels and high AP at baseline were detected in 36/86 (42%) and 18/86 (21%) of patients respectively. All patients underwent neo-adjuvant chemotherapy and surgery as per protocol. A good pathologic response (≥90% necrosis) was achieved by 45/86 (52%) of the patients.

All samples underwent decalcification as previously described [17].

For tissue microarray (TMA) construction, a slide stained with hematoxylin and eosin was prepared from each formalin-fixed, paraffin-embedded (FFPE), and representative tumor regions were morphologically identified and marked on each slide. Tissue cylinders with a diameter of 1.0 mm were punched from the marked areas of each block and brought into a recipient paraffin block. Five TMAs were constructed. Each tumor sample was represented by a minimum of 1 core to a maximum of 5 cores.

From TMA blocks, 4 micron-sections were cut. This histological sections were coloured with haematoxylin eosin and the tumor microenvironment was characterized by applying antibodies directed against fixation resistant epitopes of CD68 (tumor associated macrophages: TAM), CD3, CD8, CD20 (T tumor infiltrating lymphocytes: TILs), FOXP3 (T regulatory lymphocytes: T-regs), Tia-1 (cytotoxic T cell), BDCA-2/CD303 (plasmacytoid dendritic cells: pDC), Arginase-1 (myeloid derived suppressor cells: MDSC) proteins. PD-1 expression on immune-cells (IC), and PD-L1 on both tumour cells (TC) and IC was also investigated. The antibody reactivity, source as well as the antigen retrieval protocols were reported in Supplementary Table 1.

A semi-quantitative score from 0 to 3 was assigned to immune infiltrates: 0 = “none”: no immune infiltrates; 1 = “focal”: mostly perivascular in tumor with some intra-tumoral extension; 2 = “moderate”: prominent extension of immune infiltrates away from perivascular areas and amongst tumor cells); 3 = “severe” (immune infiltrates obscuring tumor) [4]. Pathologists were blinded to clinical information.

Only cores with tumoral component were included in the analysis. The immunehistochemical scores were generally concordant among cores of the same patient, in case of heterogeneity the highest score was considered for the analysis.

For survival analysis samples were classified as negative (immunostaining = 0) or positive (immunostaining = 1 to 3) for all markers except: CD68^+^ cases that were classified in high expressing (severe and moderate expression) and low expressing (focal expression). Score for PD-L1 expression in TC: specimens with >5% membranous expression were considered positive.

The following factors were correlated with overall survival (OS): age (pediatric < 18 years vs adult ≥ 18 years), gender, LDH and phosphatase alkaline (PA) levels at baseline (normal vs high), pathologic response (good: chemotherapy-induced tumor necrosis ≥ 90%; poor: chemotherapy-induced tumor necrosis < 90%) [17], tumoral microenvironment components, PD-1 expression on IC, and PD-L1 both on TC and IC.

OS was estimated according to the Kaplan and Meier method with their respective 95% confidence intervals (CI) and calculated from the first day of chemotherapy administration to death or last follow-up visit.

### Post-induction chemotherapy (surgical samples)

In 86/129 patients, FFPE from tumoral masses surgically resected, after neoadjuvant chemotherapy, were collected. The full slides sections from FFPA were investigate by immunohistochemistry for CD8^+^ and Tia1 expression.

The following factors were correlated with overall survival: tumoral microenvironment components (CD8 and Tia1).

## SUPPLEMENTARY MATERIALS FIGURE AND TABLE



## Abbreviations

TMAs: tissue microarrays
Pts: patients
TAM: tumor associated macrophage
pDC: plasmacytoid dendritic cells
MDSC: myeloid derived suppressor cells
IC: immune-cells
AP: alkaline phosphatase
5-year OS: 5-year overall survival
PD-1: programmed cell death 1
PD-L1: programmed cell death-ligand 1
APCs: antigen-presenting cells
