# Supplementary material for: Tumoral immune-infiltrate (IF), PD-L1 expression and role of CD8/TIA-1 lymphocytes in localized osteosarcoma patients treated within protocol ISG-OS1
Source: Oncotarget. 2017 Dec 4;8(67):111836–46. doi: 10.18632/oncotarget.22912 (PMC5762363; doi:10.18632/oncotarget.22912)
Supplement: Supplementary file 1 [file oncotarget-08-111836-s001.pdf]

Tumoral immune-infiltrate (IF), PD-L1 expression and role of CD8/TIA-1 lymphocytes in localized osteosarcoma patients treated within protocol ISG-OS1

SUPPLEMENTARY MATERIAL

Supplementary Table 1: Immunohistochemistry antibody reactivity, sources as well as the antigen retrieval protocols, dilutions and revelation systems

antibody	clone	origin	dilution	antigen retrieval	revelation system
CD3	SP7	Neomarkers	1:60	PT-link EnVision Flex	Dako Real Detection system
CD8	144B	Dako	1:100	PT-link EnVision Flex	Dako Real Detection system
CD20	L26	Dako	1:300	PT-link EnVision Flex	Dako Real Detection system
FOXP3	Sp97	Abnova	1:100	PT-link EnVision Flex	Dako Real Detection system
TIA-1	2G9	Immunotech	1:300	PT-link EnVision Flex	Dako Real Detection system
BDCA-2/CD303	124B3.13	Dendritics	1:100	PT-link EnVision Flex	Dako Real Detection system
Arginase-1	polyclonal	GeneTex	1:3200	PT-link EnVision Flex	Dako Real Detection system
PD-1	NAT-1	Prof. Roncador (Madrid)	1:4	PT-link EnVision Flex	Dako Real Detection system
PD-L1	SP263	Ventana, Roche	RTU	Ventana Cell Conditioning 1 (CC1)	Ventana OptiView Detection system
CD68	PGM1	Prof. Falini (Perugia)	1:5	PT-link EnVision Flex	Dako Real Detection system
CD163	10D6	Leica	1:100	PT-link EnVision Flex	Dako Real Detection system

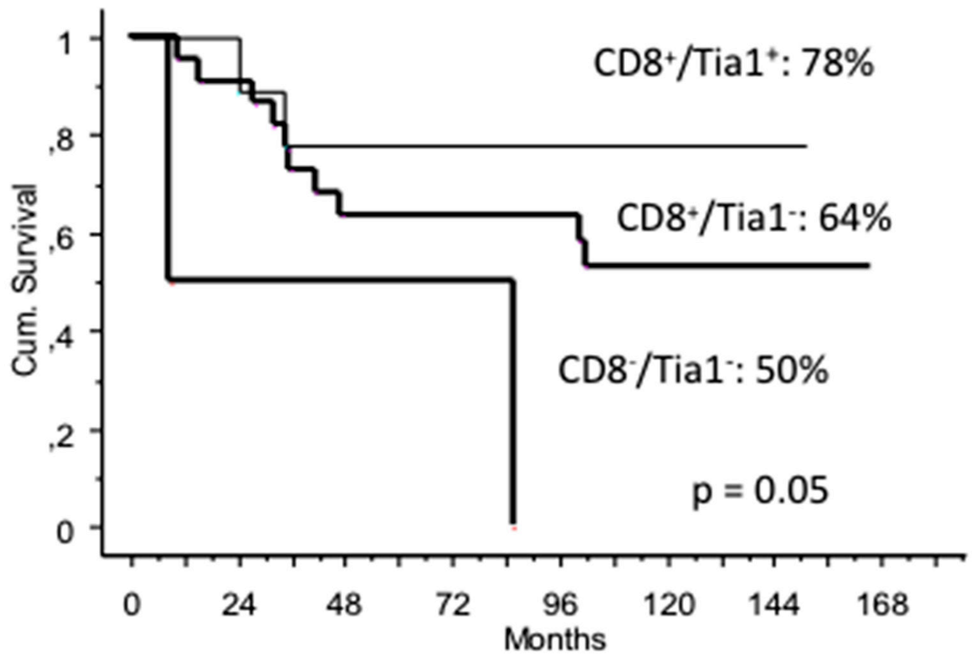

Supplementary Figure 1: 5-year overall survival according to CD8/Tia1 expression after neo-adjuvant chemotherapy in localized osteosarcoma.
